# RADIOGRAPHIC EVALUATION OF CONSERVATIVE TREATMENT OF DISTAL RADIUS FRACTURES

**DOI:** 10.1590/1413-785220243202e275070

**Published:** 2024-06-24

**Authors:** Brenno Lopes Cangussu, Henrique Cruz Baldanza, Ricardo Leão Carmo, Daniel Magalhães Nobre, Alexandre Ramos Estanislau, Tomás Santos Vasconcelos Barros

**Affiliations:** 1 Hospital Municipal Odilon Behrens, Department of Orthopedics, Belo Horizonte, MG, Brazil.; 2 Hospital Municipal Odilon Behrens, Teaching and Research Committee, Belo Horizonte, MG, Brazil.

**Keywords:** Fracture, Distal Radius, Conservative Treatment, Radiography, Fraturas Distais do Rádio, Tratamento Conservador, Radiografia

## Abstract

**Objective:**

This article aims to evaluate the evolution of radio-graphic parameters (radial tilt, volar tilt, and radial height) of distal radius fractures in patients indicated for conservative treatment at three different times: date of diagnosis, first outpatient visit within 2 weeks after closed reduction, and last outpatient visit.

**Methods:**

We included 84 patients seen at the emergency department of Hospital Municipal Odilon Behrens, with a diagnosis of distal radius fracture and an indication for conservative treatment. We considered only those patients who had serial radiographs taken at least three different times (n=69) in this analysis.

**Results:**

There was an improvement in radiographic parameters of volar tilt after closed reduction and immobilization, which was maintained until the last outpatient visit. Radial inclination and radial height showed increased values from the first to the second radiographic evaluation and both values had regression when comparing the second to the third (last) evaluation.

**Conclusion:**

Universal classification stable fractures tend to evolve well with conservative therapy. ***Level of Evidence II; Development of Diagnostic Criteria in Consecutive Patients (with Gold Standard of Reference Applied).***

## RESUMO

**Objetivo:**

O objetivo deste artigo foi avaliar a evolução dos parâmetros radiográficos (inclinação radial, inclinação volar e altura radial) das fraturas da extremidade distal do rádio em pacientes com indicação de tratamento conservador em três momentos diferentes: data do diagnóstico, primeira consulta ambulatorial dentro de. semanas após a redução fechada e última consulta ambulatorial.

**Métodos:**

Incluímos 84 pacientes atendidos no departamento de emergência do Hospital Municipal Odilon Behrens, com diagnóstico de fratura distal do rádio e indicação de. conservador. Consideramos nesta análise apenas os pacientes que tiveram radiografias seriadas realizadas pelo menos três vezes diferentes (n=69).

**Resultados:**

Houve uma melhora nos parâmetros radiográficos da inclinação volar após a redução fechada e a imobilização, que foi mantida até a última consulta ambulatorial. A inclinação radial e a altura radial apresentaram valores aumentados da primeira para a segunda avaliação radiográfica e ambos os valores tiveram regressão quando comparados da segunda para a terceira (última) avaliação.

**Conclusão:**

As fraturas estáveis de classificação universal tendem a evoluir bem com a terapia conservadora. ***Nível de Evidência II; Desenvolvimento de critérios diagnósticos em pacientes consecutivos (com aplicação de referência padrão ouro).***

## INTRODUCTION

Defined as those that occur up to 3 cm from the radius carpal joint, fractures of the distal end of the radius account for 17% of all fractures seen in emergency rooms.^1^ Middle-aged women are the most affected, with a significant increase in incidence over the age of 50. ^2^

In order to define the best type treatment it is fundamental to initially classify them in relation to the parameters of instability, reducibility, the fracture mechanism and associated injuries, as well as the patient's age and comorbidities.^2^

Among the classifications most widely used today, we highlight three that have greater practical applicability because they provide contributions to the treatment and prognosis of fractures: the AO classification, the Universal classification described by Raylock in 1990, modified by Cooney in 1993, and the Fernandez classification, described in 1994 (Board 1, 2 and 3).

**Board 1:** Universal Classification (COONEY).

Universal Classification (COONEY)
I. Extra-articular without deviation
II. Extra-articular with deviation
A. Stable reducible
B. Unstable reducible
C. Irreducible
III. Intra-articular without deviation
IV. Intra-articular with deviation
A. Stable reducible
B. Unstable reducible
C. Irreducible
D. Complex

The Universal classification described by Raylock in 1990, modified by Cooney in 1993.

**Board 2:** Fernandez Classification.

I. fractures produced by angulation of the distal metaphysis of the radius, in which a cortical suffers a degree of comminution, as seen in Colles' and Smith's fractures;
II. fractures produced by shear mechanism, such as Barton's fractures and those of the radial styloid apophysis;
III. compression of the articular surface with impaction of the subchondral bone and the epiphysis. Current terms used for this fracture are complex articular fracture or radial pilon fracture;
IV. avulsion mechanism, includes radial and ulnar styloid fractures associated with carpal displacement;
V. fractures produced by high-energy trauma, translated by the combination of the four previous mechanisms, that means, angulation, shear, compression, and avulsion.

**Board 3:** AO Classification – Distal Radius Fractures.

AO classification - distal radius - 2R3
Extra articular - 2R3A
Radial styloid avulsion - 2R3A1
Single line - 2R3A2
Wedge or multifragment 2R3A3
Partial articular - 2R3B
Sagittal 2R3B1
(Barton) dorsal rim 2R3B2
(Reverse Barton) volar rim2R3B3
Complete articular 2R3C
Simple articular and metaphysis - 2R3C1
Multifragmentary Metaphyseal - 2R3C2
Multifragmentary articular, simple or multifragmentary metaphyseal - 2R3C3

AO/OTA international classification of fractures and dislocations.

In a conservative approach, closed reduction of the fracture is performed when necessary and cast immobilization for approximately six weeks. The objective of this intervention is the anatomical restructuring and return of its functionality as close as possible to the physiological one.^3^

This study, carried out at Hospital Municipal Odilon Behrens - Belo Horizonte - MG, was performed through periodic patient follow-up at the orthopedics/hand surgery outpatient clinic. Radiographic parameters in distal radius fractures were evaluated.

## MATERIALS AND METHODS

The study included 84 patients who sought emergency care at the Odilon Behrens Municipal Hospital and whose diagnosis was distal radius fracture. After diagnosis and conservative treatment of the fractures, the patients were followed up as outpatients, performing radiographic control.

Cooney's Universal Classification (Board 1) was used to evaluate the fractures and plan inclusion or exclusion of patients in the study, by means of radiographic criteria.

For analysis in our study, only patients who had undergone out-patient follow-up in our service, with radiographic control in at least three distinct times (n=69), such as: day of diagnosis and immobilization, first outpatient visit (two weeks) and last visit.

There were excluded patients who had deviated and irreducible extra-articular (Cooney IIC), deviated and irreducible articular (Cooney IVC), complex fractures (Cooney IVD), and fractures with more than two criteria of instabilities defined by Lafontaine (Board 4). There were excluded patients who did not have adequate follow-up, did not have radiographic control or did not attend return visits. Two patients who were referred to surgery after two weeks of immobilization due to fracture deviation, were excluded from the study. These two were previously classified as Cooney IVB (intra-articular with deviation and unstable reducible).

**Board 4:** Lafontaine Criteria.

Lafontaine Criteria
Dorsal deviation > 20°, dorsal comminution, radial shortening > 9 mm, radiocarpal and distal radioulnar articular involvement, associated ulnar fractures, intra-articular fragment spacing > 2 mm, and age > 60 years.

Criteria of instabilities defined by Lafontaine in 1989. Type 1: 0-1 criterion (stable); Type 2: 2-3 criteria (potentially unstable); Type 3: ≥4 (unstable).

We take into account three radiographic parameters: radial tilt/ulnar tilt of the radius, volar tilt, and radial height.

All patients in the study were made aware of the risks and benefits of participating in the research through the Free and Informed Consent Form (TCLE), having signed this document.

The present study was approved by the ethics and research committee through the brasil platform under number 5.113.627.

## RESULTS

Out of the total 84 patients initially selected in the study, 55 (65%) participants required closed reduction in the emergency room.

Most patients (n=68; 81%) used axillopalmar cast, 10 patients (12%) used antebrachiopalmar cast, 2 patients (2%) used long splint and 2 patients (2%) used short splint immobilization. Out of the total 69 patients included in the study 54 were women (78%), 47 patients were aged 50 years or older (68.11%).

The patients had UNIVERSAL classification information at the date of diagnosis (pre-reduction). Out of this total, 14.2% were classified as Cooney I; 19% Cooney IIA, 8.3% Cooney IIB, 10.7% Cooney III, 4.7% Cooney IVA, 8.3% Cooney IVB.

**Figure 1:**
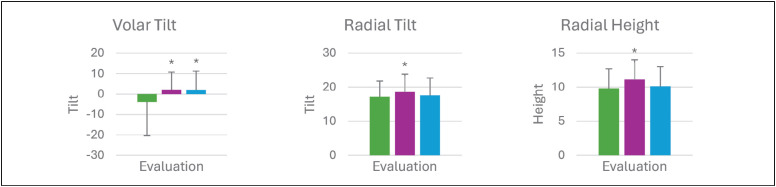
Changes shown by each variable in the three evaluation moments.

It was observed a significant increase in volar tilt values during the evaluations, after closed reduction, immobilization, and a new radiography in a return visit (-3.8°, 2.0°) P<0.001. (Table 1) The radial tilt and radial height showed increased values from the first to the second radiographic evaluation. Both values had regression when comparing the second to the third (last) evaluation (17.2° / 18.6° / 17.6°) P<0.001, (9.8 mm/ 11.1 mm/ 10.1 mm) P<0.001. (Figure 1) Data presented on average (standard deviation). * statistically significant difference compared to the first evaluation in the One-Way ANOVA test (p<0.05).

**Table 1:** Variables with the changes presented by each variable in the three evaluation moments.

Variable	1º	2ª	3ª	F-value	p-value	n
Volar	-3,8 (16,6)	2,1 (8,6)*	2,0 (9,2) *	10,92	<0,001	0.05 (Small)
Radial	17,2 (4,6)	18,6 (5,2)*	17,6 (5,1)	6,723	<0,001	0.01 (Small)
Radio	9,8 (2,9)	11,1 (2,9)*	10,1 (2,9)	11,45	<0,001	0.03 (Small)

Data presented on average (standard deviation).

* statistically significant difference compared to the first evaluation in the One-Way ANOVA test (p<0.05).

## DISCUSSION

Wrist fractures involving the distal radius are frequent injuries in emergency care, affecting mostly middle-aged women. With n=54 (78%), they were also predominant in our study.^4^

With this research it was possible to observe through radiographic criteria, the evolution of the non-surgical treatment of distal radius fractures, using the criteria cited previously.

From the 3 different forms of immobilization (axillopalmar circular cast, antebrachiopalmar circular cast, and short plaster cast), no significant discrepancy was observed in the outcome (loss of measurements obtained in closed reduction at the first and third visits). However, although this was not a randomized study, it was observed a tendency of the use of short splint/plaster for reducible and stable fractures.^5-7^

In this study, there was an improvement trend in the radiographic parameters from the first to the second moment (after reduction), followed by a slight loss of the values obtained when analyzed at the third moment.

The measurements and angulations of the distal radius are predictors of success in choosing non-surgical treatment. They are: radial height, volar tilt, and radial slope. In a treatment that can vary from 4 to 8 weeks, these values of measurements and angulations classically are responsible for defining the continuation or discontinuation of the therapeutic method, resulting in maintenance of reduction or loss of fracture reduction.^8^

Radiographic parameters such as volar tilt and radial height correlate closely with clinical outcomes in conservative therapy, so they must be carefully evaluated at well-defined intervals.^9^

Most distal radius fractures can be treated with non-surgical therapies, even if it is only the first choice. If after a period of radiographic control it is possible to find other treatment options. The exception is for comminuted, intra-articular fractures, and those with significant deviation. For example, 2 patients who were excluded from the study due to a change in treatment: after a conservative approach, were referred to surgery for deviation of a fracture after two weeks of cast immobilization.^10^

A limiting factor in the study concerns the radiographic indicators, although commonly used, there is no consensus or well-established guidelines as to which descriptors should actually be used and how they should be performed to guide the course and choice for conservative therapy.^2^

It was possible to analyze the primary outcome: analysis of the radiographic parameters radial height, radial tilt and volar tilt, which have a strong relationship with the primary outcomes.

## CONCLUSION

The good follow-up of the conservative treatment is closely linked to a good technique of closed reduction and immobilization with a correctly positioned external imobilization, either short/long or splint. Stable fractures by the Universal classification tend to evolve well with conservative therapy. A change of treatment to the surgical option was observed in two patients who initially had a classification (intra-articular BVI with deviation and unstable reducible).

Indicators such as volar tilt, radial tilt and radial height were the points of important radiographic analysis and divided into three moments, making it possible to infer the evolution of the non-surgical management and predict the clinical results of the conservative choice.
